# A Polymeric Composite Material (rGO/PANI) for Acid Blue 129 Adsorption

**DOI:** 10.3390/polym12051051

**Published:** 2020-05-03

**Authors:** Tomasz Kukulski, Stanisław Wacławek, Daniele Silvestri, Kamil Krawczyk, Vinod V. T. Padil, Ryszard Fryczkowski, Jarosław Janicki, Miroslav Černík

**Affiliations:** 1 Institute of Textile Engineering and Polymer Materials, University of Bielsko-Biala, Willowa 2, 43-309 Bielsko-Biala, Poland; tkukulski@ath.bielsko.pl (T.K.); rfryczkowski@ath.bielsko.pl (R.F.); jjanicki@ath.bielsko.pl (J.J.); 2 Institute for Nanomaterials, Advanced Technologies and Innovation, Technical University of Liberec, Studentská 1402/2, 46117 Liberec 1, Czech Republic; kamil.krawczyk@tul.cz (K.K.); vinod.padil@tul.cz (V.V.T.P.); miroslav.cernik@tul.cz (M.Č.)

**Keywords:** graphene, nanocomposite, adsorption, Acid Blue 129

## Abstract

Over the years, polyaniline (PANI) has received enormous attention due to its unique properties. Herein, it was chosen to develop a new polymeric composite material: reduced graphene oxide/polyaniline (rGO/PANI). The composite was prepared by a simple and cost-effective fabrication method of formation by mixing and sonication in various conditions. The obtained materials were characterized and identified using various techniques such as scanning electron microscopy (SEM), Raman and ATR–FTIR spectroscopy, and X-ray diffraction (XRD). The objective of the paper was to confirm its applicability for the removal of contaminants from water. Water could be contaminated by various types of pollutants, e.g., inorganics, heavy metals, and many other industrial compounds, including dyes. We confirmed that the Acid Blue 129 dyes can be substantially removed through adsorption on prepared rGO/PANI. The adsorption kinetic data were modeled using the pseudo-first-order and pseudo-second-order models and the adsorption isotherm model was identified.

## 1. Introduction

Recently, graphene has been considered as a wonder material, especially after Prof. Geim and Novoselov gained Nobel prize for obtaining its stable form in 2010 [1]. They used mechanical exfoliation named today the ‘Scotch tape method’, which is considered as one of the physical ‘top-down’ approaches to get nanolayers. The other interesting way of obtaining graphene is by chemical methods. The first documented approach was made by Brodie in 1858 [2], whereas the next one by Staudenmaier in 1899 [3] and Hummers in 1958 [4], which nowadays is the most famous method for synthesis of graphene or its derivatives graphene oxides. Moreover, many modifications of this approach exist, for example Tour or Shi methods [5,6]. All the chemical methods are based on graphite oxidation by a strong oxidizer such as KMnO_4_ or KClO_3_, when graphene oxide (GO) is produced. It can be further deoxidized to reduced graphene oxide (rGO) by many reduction methods, such as chemical (e.g., hydrazine) [7,8], electrochemical [9], or thermal [10]. Graphene has extraordinary physical properties, owing to which, it has many potential applications in electronics, optics (as sensors), or as a component used in membranes and batteries [11,12,13,14,15,16]. Indeed, water treatment is a growing field for graphene-based materials [17,18,19,20,21]. 

Another interesting material that has recently brought the scientist´s attention (even though it was discovered over 150 years ago) is polyaniline (PANI) [22]. It is a conducting polymer with many advantages, e.g., simple synthesis, low cost, stability in environment, and easy doping/dedoping chemistry [23,24,25]. According to this it is used as protective coatings or supercapacitors [26,27]. These two remarkable materials can be bonded together to form the rGO/PANI composite. This composite is used often in supercapacitors applications [28]; however, recently it has been proposed as an excellent adsorbent [29], e.g. for heavy metals and dyes adsorption [30,31,32]. rGO due to the large specific surface area and PANI which is known for its facile synthesis, insolubility in water and stability, are potentially very good sorption materials [33,34]. The wastewater from the dyeing industry has many toxic properties and it has to be treated before the release to the environment [35]. Water and wastewater treatment are considered a vital branch of the environmental chemistry [36,37].

In this work we believed that the herein synthesized rGO/PANI composite can be used for efficient removal of the Acid Blue 129 (AB129) from water. The rGO/PANI composite was made by sonication and mixing at different temperatures. Taking into account the importance of the development of novel water treatment technologies and after successful trials for the removal of toxic metals from water by rGO/PANI composite [30], we are demonstrating herein first use of rGO/PANI for the adsorption of anionic dye from water. This study gives additional evidence of the possibility of using rGO/PANI for water and wastewater treatment.

## 2. Materials and Methods

### 2.1. Chemicals

Graphite (particle size <20 µm), polyaniline (PANI; emeraldine base, average molecular weight ~50,000), Acid Blue 129 (dye content 25%), hydrochloric acid (35 wt %), sulfuric acid (96 wt %), potassium permanganate (99.5 wt %), and hydrogen peroxide (30 wt %) were purchased from Sigma Aldrich (Saint Louis, MO, USA). Deionized water (18.2 MΩ·cm) was obtained by an ELGA purelab flex system (ELGA, Veolia Water, Marlow, UK) and was used in all experiments.

### 2.2. Preparation of Reduced Graphene Oxide

GO was synthesized by the modified Hummers’ method described before [38]. Briefly, 0.5 L of H_2_SO_4_ was poured into a 5 L beaker and then 20 g of graphite was added. The mixture was stirred on a magnetic stirrer for 1 h, and then cooled down in an ice bath to 5 °C. After, 60 g of KMnO_4_ was added in small batches, so the temperature of the reaction mixture would not exceed 35 °C. After adding KMnO_4_, the reaction mixture was allowed to rest for 2 h, while the temperature was constantly controlled to keep it under 50 °C. In a subsequent step, 1 L of distilled water was added to the reaction mixture also in small batches, so the temperature of the reaction mixture was kept below 65 °C. Then, 0.8 L of distilled water with a temperature of 60 °C and 0.8 L of 3% H_2_O_2_ were added. GO was purified with 10% HCl and distilled water and finally centrifuged. Pure graphene oxide after drying was thermally reduced at 500 °C and nitrogen atmosphere.

### 2.3. Preparation of rGO/PANI Nanocomposite

There are only few works, which report green and effective sono-assisted synthesis of rGO-PANI composite [39,40,41]. In this work, we have extended these investigations to show the effect of various conditions on rGO bonding with PANI. PANI (3.88 g) was dispersed in 100 mL solution of 1 M HCl. Afterwards 0.5 g of rGO was added to this mixture with subsequent sonication for 15 mins. Thus created dispersions were mixed on a magnetic stirrer in various temperatures and periods (samples are named rGO/PANI-x,y; where x is temperature and y is time period, Table 1). The (expected) formation of amide bonds between rGO and PANI (as well as other chemical reactions), are time and temperature dependent. Therefore, the main aim of this part of the study was to investigate their influence on the rGO and PANI conjugation through the amide bond formation. The temperature range chosen and synthesis time were in accordance with the study of Sibilska et al. [42].

After mixing, samples in the form of powder were filtered under vacuum and purified by distilled water and 100 mL of 0.1 M NH_3_ (two times) to reach neutral pH, in order to reach the PANI in the form of emeraldine base.

### 2.4. Characterization Procedures

SEM (Phenom ProX, Thermo Scientific, Waltham, MA, USA) operating at an acceleration voltage 10 kV was used to analyze morphology and the structure of the composites. Before SEM analysis the samples were set onto aluminum holders, then, the sample holders were diffusion-coated with thin gold layer. Raman scattering was performed on micro Raman spectrometer (Raman DXR microscope, Thermo Scientific, Waltham, MA, USA) at 514 nm laser excitation of an argon laser with a spectral resolution of 1 cm^−1^ (full-width at half-maximum). XRD analysis measurements were taken using a URD 63 diffractometer (FPM-Seifert, Hamburg & Freiberg, Germany). The Cu Kα radiation was used at 40 kV and 30 mA. The monochromatization of the beam was achieved by a pulse height analyzer and nickel filter. Scintillation counter served as a detector. The searches were carried out in the range of angles from 4° to 60° at 0.1° pitch. Each diffraction curve was corrected for polarization, Lorentz factor and incoherent scattering. Attenuated total reflection—Fourier transform infrared spectroscopy (ATR–FTIR) spectra were obtained at 4000–7000 cm^−1^ (4 cm^−1^ resolution) utilizing a germanium ATR crystal (NICOLET IZ10, Thermo Scientific, Waltham, MA, USA) equipped with a horizontal ATR accessory (single reflection angle 45°). The surface areas of the composites were obtained using the BET (Brunauer−Emmett−Teller) technique (Autosorb iQ, Quantachrome Instruments, FL, USA). Absorption spectra were analyzed in a UV–vis spectrophotometer (Hach Lange DR 3900, Vancouver, WA, USA).

### 2.5. Quantum Chemical Analysis

The initial coordinates of the rGO/PANI composite were obtained with the Avogadro program (Open Molecules, Pittsburgh, PA, USA) [43]. All of the calculations were made with the Gaussian 16 software (Gaussian Inc. Wallingford CT, USA) [44]. The B3LYP/6-31G level of study was employed. Aniso-surface threshold value of 0.01 atomic units was set. The outputs were visualized with the Avogadro program.

### 2.6. Acid Blue 129 Adsorption Experiments

Adsorption tests were carried out in 100 mL beakers (50 mL dispersion of 25 mg rGO/PANI composite). The AB129 solutions (25 mg/L) were prepared by the addition of dye to the DI water. Samples were centrifuged at 14500 RPM and measured by the spectrophotometric technique with UV–vis spectrophotometer (Hach Lange DR 3900, Vancouver, WA, USA). Kinetic experiments were performed with all samples at room temperature and for 60 mins. 

Kinetics models were used to determine the adsorption controlling mechanism, based on the experimental data. The pseudo-first-order kinetic model described the rate of the adsorption based on the adsorbed amounts. Its linear form is usually expressed as (Equation (1))

(1) $$log(q_{e}-q_{t})=logq_{e}-\frac{k_{1}}{2.303}t,$$

where *q*_e_ and *q*_t_ are the adsorption amounts at equilibrium and time *t*, respectively, and *k*_1_ is the first-order rate constant. A plot of log (*q*_e_ − *q*_t_) versus *t* gives *k*_1_ as the slope and log *q*_e_ as the intercept value.

The pseudo-second-order kinetic model employed to adsorption kinetics was presented in [45] as

(2) $$\frac{t}{q_{t}}=\frac{1}{k_{2}q_{e}^{2}}+\frac{1}{q_{e}}t,$$

where *k*_2_ is the appropriate rate constant. The plot of *t*/*q*_t_ versus *t* shows a linear relationship if the second-order kinetic is appropriate. Values of *k*_2_ and *q*_e_ were calculated from the intercept and slope of the plots.

Langmuir adsorption isotherms were calculated based on batch tests according to equation

(3) $$q_{e}=\frac{q_{max}k_{l}C_{e}}{1+k_{l}C_{e}}$$

where *q*_e_ and *q*_max_ is the equilibrium and maximum adsorption amount, respectively, *C*_e_ the equilibrium concentration in solution and *k_l_* is the Langmuir isotherm equilibrium constant related to free energy of adsorption. The adsorption was studied at a composite concentration of 0.5 g/L and different initial dye concentrations (3.1–50 mg/L). 

The slope of the plot of *C*_e_/*q*_e_ against *C*_e_ gives slope 1/*q*_max_, the intercept 1/*q*_max_*k*_l_. The coherence between obtained results and the model-predicted values is indicated by the R^2^ (determination coefficient).

## 3. Results and Discussion

### 3.1. Characterization

#### 3.1.1. XRD

**Figure 1:**
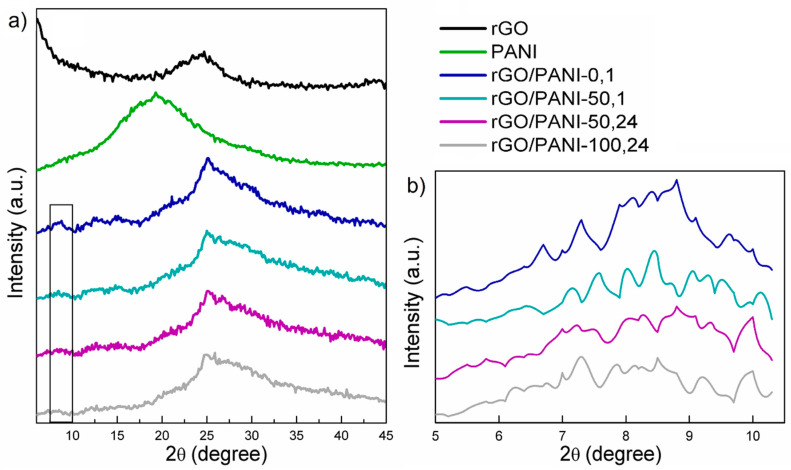
(**a**) Results of XRD diffractograms of (from the top) rGO, PANI; rGO/PANI-0,1; rGO/PANI-50,1; rGO/PANI-50,24 and rGO/PANI-100,24 samples. (**b**) Magnified view of the 2θ = 7°–8° peak.

In Figure 1, XRD patterns of the rGO, PANI, and composites samples can be observed.

As it could be seen, the XRD patterns of the pure rGO (Figure 1a) exhibit one typical peak at 2θ = 24° [46]. As for the bare PANI sample, one broad peak at 2θ = 20° could be observed. The XRD pattern of the rGO/PANI composite indicates a protonation of PANI. This could be evidenced by the shifting and division of the original PANI peak at 2θ = 20° to two peaks at 25° and 30°(Figure 1a). Moreover, in the same spectrum, a new peak appears at 7°–8° and the share of the amorphous component grows, it is visible as a broad halo around 30° [47,48]. According to these results, we could speculate about permanent bonding between the rGO and PANI. This could be further confirmed by the ATR–FTIR results presented in the subsequent subsection.

Furthermore, in Figure 1b comparison of the XRD spectra in the low-angle region was shown. The peak at the angle of 2θ = 7°–8° could be correlated to the more uniform dispersion of PANI on rGO [49] as well as to the protonation level of PANI (higher protonation state relating to a higher peak intensity [47]). From this, it can be concluded that PANI not only organizes itself under the influence of rGO but also the protonated = NH^+^– groups can attract and immobilize negatively charged species [50], such as anionic dyes. Therefore, rGO-PANI-0,1 sample, having the highest intensity of 2θ = 7°–8° peak is believed to also have high potential of adsorbing Acid Blue 129 as well as other sulfonic/anionic dyes. However, in order to confirm this statement, it had to be validated whether PANI is undeniably covalently bound to rGO. This was assessed by ATR–FTIR and discussed in the next subsection.

#### 3.1.2. ATR–FTIR

**Figure 2:**
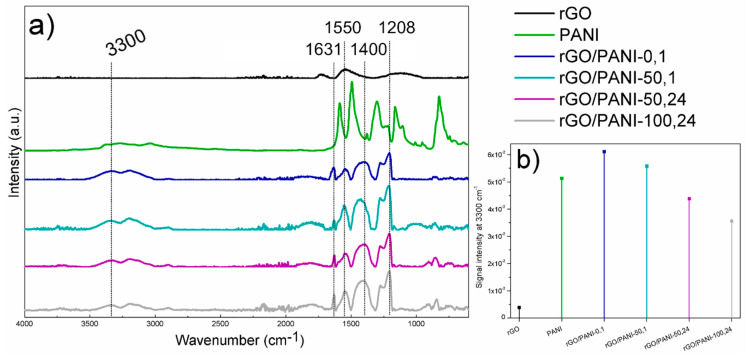
(**a**) Results of ATR–FTIR analysis of (from the top) rGO; PANI; rGO/PANI-0,1; rGO/PANI-50,1; rGO/PANI-50,24 and rGO/PANI-100,24 samples. (**b**) Comparison of peak intensities at 3300 cm^−1^.

ATR–FTIR analysis was performed to confirm functional groups (and changes) present in PANI, rGO and the composite (Figure 2). This method is suitable for observing materials showing high absorption in the infrared range as the tested materials.

The rGO spectrum shows residual oxygen group remaining after reduction at the wavenumber of 1730 and 1130 cm^−1^, C=O (carbonyl group) and C–OH, respectively. The peak at 1530 cm^−1^ corresponds to stretching C=C. The PANI spectrum shows characteristic peaks from benzenoid rings at 1587 and 1494 cm^−1^, C–N band of an aromatic amine and N=Q=N vibration (Q- quinoid ring) at 1301 and 1166 cm^−1^, respectively.

After the reaction of rGO with PANI, the peaks from benzenoid rings shift to the lower energy at 1629 and 1546 cm^−1^, whereas at 1400 cm^−1^ a new peak characteristic for amide C–N bands appeared with a simultaneous signal increase typical for N–H stretching vibrations in a secondary amide (Figure 2b) at 3300 cm^–1^ [51]. Moreover, the peak present in the rGO/PANI spectrum at 1207 cm^−1^ is also characteristic for C–N stretching. The observed bands confirm the formation of chemical bonds between PANI and rGO. These types of interactions have a decisive impact on the electronic structure of the resulting composite, but also affect the supramolecular structure which has a significant impact on sorption properties, as well as the adsorption model of ionic compounds.

Due to the highest intensity of the peak at 3300 cm^−1^ and at 7°–8° given by FTIR and XRD analyses respectively, and the time spent for sample preparation (only 1 h of synthesis in a low temperature), rGO/PANI-0,1 sample has been selected for further investigations.

#### 3.1.3. Morphology and Electron Distribution

**Figure 3:**
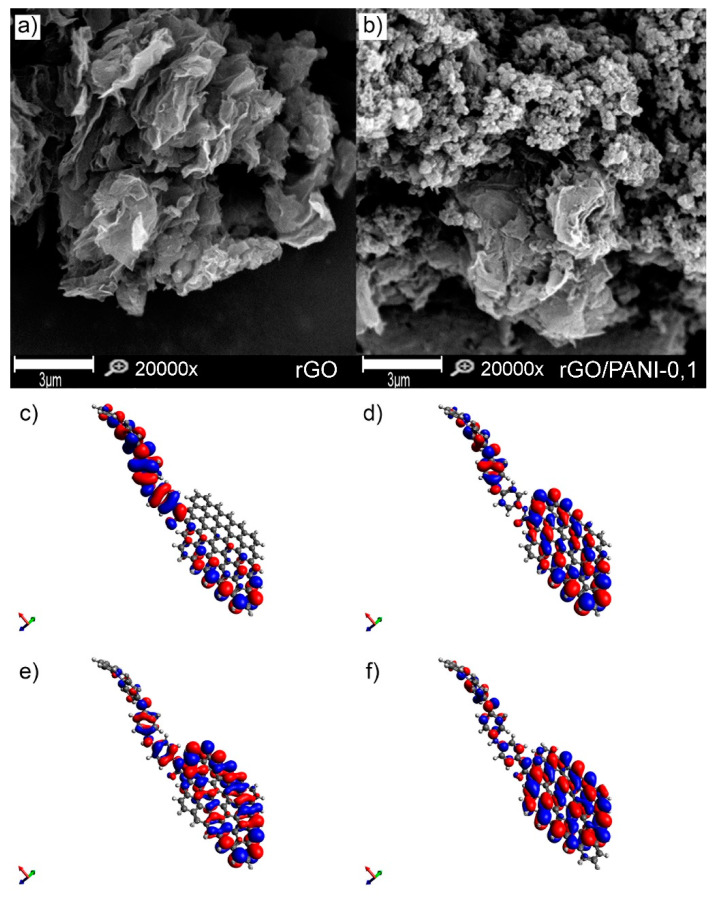
SEM images of (**a**) rGO, (**b**) rGO/PANI-0,1 composite (scale bar represents 3 µm), (**c**) LUMO, (**d**) HOMO, (**e**) LUMO-1, and (**f**) HOMO-1 (c, d, e and f figures are not in scale).

The morphology of rGO and rGO/PANI composite is shown in Figure 3a,b, respectively.

It is visible that the pure rGO sample is composed of the typical graphene sheets. Plates of rGO have a clean and smooth surface. After mixing rGO with PANI, a composite was form, which comprised of rGO flakes covered by PANI aggregated spheres (Figure S1) with an extensive specific surface (Figure 3b). Composites have a similar morphology regardless of the conditions of formation (Figure S2). A similar structure of the composite rGO/PANI was observed by Yang et al. [52], who has synthesized it for supercapacitors application. However, in the reported method herein, PANI with a higher molecular weight was used, which can affect the adsorption and aggregation process.

Moreover, for better visualization of the rGO/PANI, a model of this composite was created with its electron distribution computed (Figure 3c–f). The lowest unoccupied molecular orbitals (LUMO) and highest occupied molecular orbitals (HOMO) of the rGO/PANI (Figure 3c,d) demonstrate large pi-pi conjugated system of connected by amide bond PANI molecule with the reduced graphene oxide. Moreover, as shown in Figure 3e,f, the extended pi-pi conjugation in the entire system was also confirmed by the LUMO-1 and the HOMO-1, indicating that amide groups play a significant role in bridging these two components [40]. Such extended system can be beneficial for adsorption of, e.g., contaminants with aromatic groups as reported by Peng et al. [53].

#### 3.1.4. Raman Analysis

**Figure 4:**
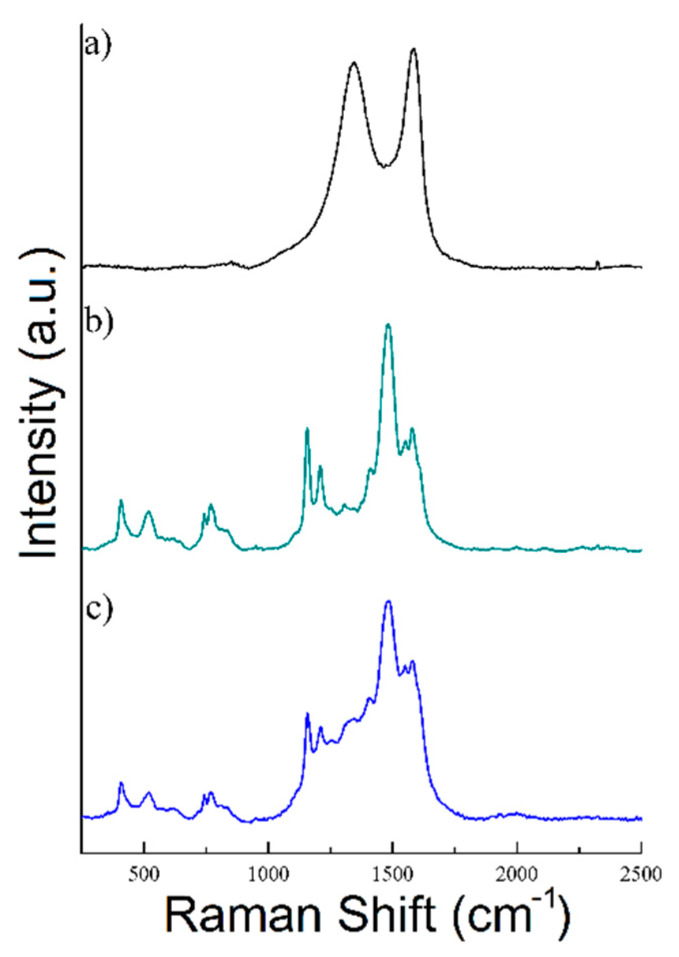
Raman spectra of (**a**) rGO, (**b**) PANI, and (**c**) rGO/PANI-0,1 composite.

Figure 4a–c present the Raman spectra of rGO, PANI and rGO/PANI, respectively.

As shown in Figure 4a rGO sample exhibits a typical Raman spectrum composed of two characteristic peaks D (at 1344 cm^−1^) and G (at 1582 cm^−1^) [54,55]. Moreover, the ratio of *I*_D_/*I*_G_ bands was determined to be 0.94 in this sample, which is a typical value for rGO [56,57,58,59]. Nonetheless, the spectrum of PANI has shown C=C stretching in the quinonoid ring at 1548 cm^−1^, C=N stretching vibration at 1483 cm^−1^, C–N stretching vibrations of diverse benzenoid at 1210 cm^−1^ and C–H bending of the quinonoid ring peak at 1158 cm^−1^. rGO/PANI composite has shown peaks at 1210, 1338, 1488, 1548, and 1583 cm^−1^ which could be found in the materials characterized individually. The 1483 cm^−1^ band shift may indicate a change in the area of the C=N bonds in PANI, which confirms the formation of the composite.

### 3.2. Adsorption of a Model Dye

AB129 was used as a model dye pollutant to assess rGO/PANI adsorption properties. After the composite was exposed to AB129, the blue color of the original solution slowly diminished. Typical Vis peak of AB129 at 630 nm wavelength progressively decreased over time. Furthermore, the rGO/PANI composite became blue due to the dye adsorption onto the composite surface. To characterize the interaction of the dye with adsorbents, important parameters must be determined such as adsorption capacity, isotherms, kinetics.

**Figure 5:**
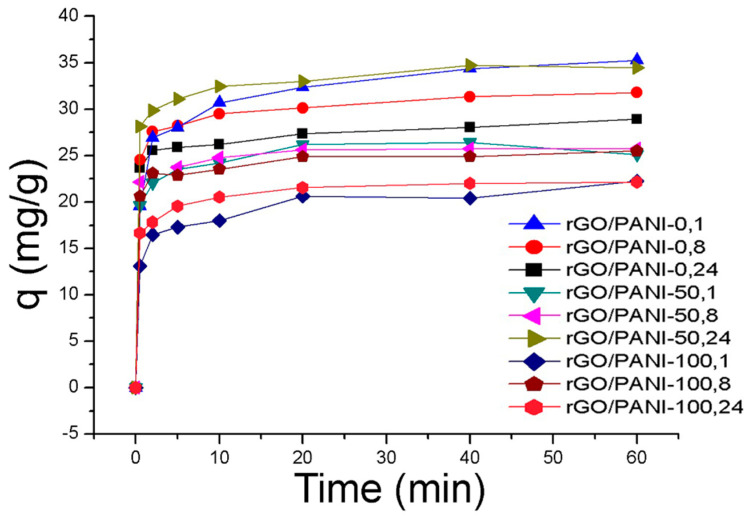
Adsorption of AB129 on rGO/PANI composites (conditions: 25 mg/L of AB129, 25 mg/50 mL of rGO/PANI, room temperature), the error bar was <5%.

Adsorption experiment was carried out in a given ratio of the composite in water under room temperature for one hour. Adsorption kinetic for all prepared samples of the composite and the AB129 dye is shown in Figure 5.

**Figure 6:**
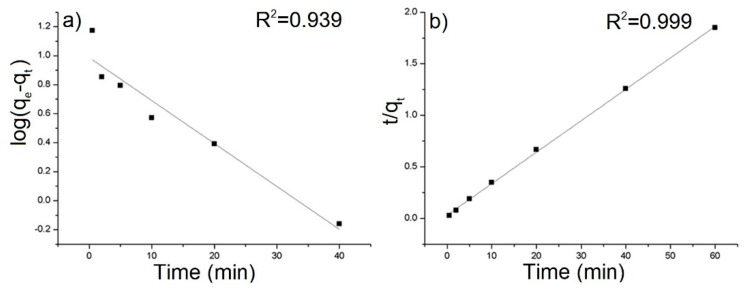
(**a**) Pseudo-first-order and (**b**) pseudo-second-order kinetic model for the adsorption of AB129 on rGO/PANI-0,1. The solid lines present fits of the pseudo-first and pseudo-second order kinetic model (conditions: 25 mg/L of AB129, 25 mg/50 mL of rGO/PANI, room temperature).

The equilibrium sorption capacity, *q*_e_ was obtained in 40 mins. The results show that the equilibrium adsorbed concentration *q*_e_ of AB129 range from 22.15 to 35.26 mg/g depending on type of composite. The lower values were reached by samples, which were synthesized at a higher temperature. The best result of *q*_e_ was obtained by the rGO/PANI-0,1 composite (Figure 5) and therefore all further studies were performed only for this sample. To obtain the information on the adsorption rate and mechanism, adsorption kinetics were fitted with both pseudo-first and pseudo-second kinetic adsorption model (Figure 6). The R^2^ of the pseudo-second-order model (R^2^ = 0.999) is higher than one for the pseudo-first-order model (R^2^ = 0.939) which is in consensus with previous observations [60]. This result was also checked on the other composite samples, where in most cases the pseudo-second kinetic adsorption model fitted the data better (Table S1). This model also includes chemisorption which may be a rate-limiting step [61]. 

**Figure 7:**
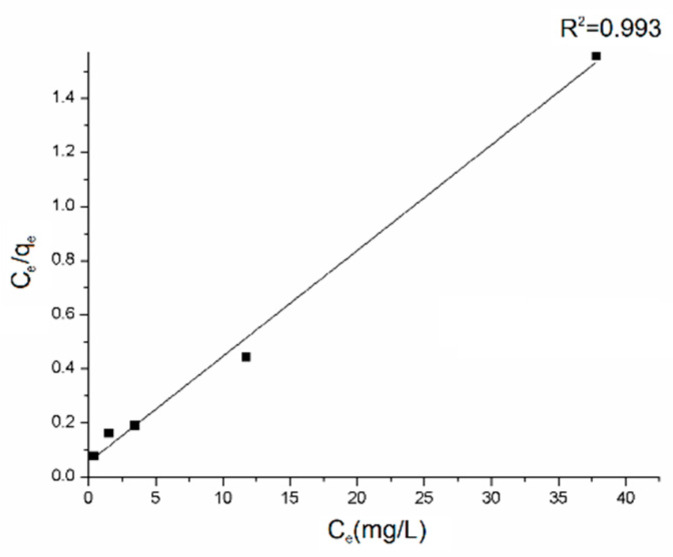
Langmuir isotherm model plot for the adsorption on rGO/PANI-0,1 (contact time: 60 mins).

Equilibrium adsorption data determined for five different initial concentrations of dye were linearized to get parameters of Langmuir isotherm [62] (Figure 7). 

The Langmuir isotherm is a satisfactory model for describing the correlation between the amounts of AB129 adsorbed by the composite and its equilibrium concentration in the solution. The maximum adsorption capacity (*q*_max_) was calculated to be 25.57 mg/g, based on the fitting result where R^2^ was 0.975. The main mechanisms involved in the adsorption of dyes onto the rGO/PANI composites as reported in many other studies are electrostatic attraction, physisorption, and complexation [29].

The obtained results are presented in Table 2 and were compared with the materials reported in the literature. In comparison to the other materials, rGO/PANI-0,1 composite is fast and effective adsorbent for AB129 removal from aqueous solution.

## 4. Conclusions

In this research, rGO/PANI composites were determined to be effective adsorbers for Acid Blue 129 dye. The composites were successfully prepared by a novel way relying on mechanical mixing at various times and temperatures. A total of nine composites were tested, from which the rGO/PANI-0,1 composite (synthesized in the lowest temperature) exhibited superior supramolecular and chemical structure that improved its adsorption properties.

SEM analysis shown structure of the composite, in which PANI particles were distributed on the rGO surface. Raman and ATR–FTIR spectroscopy confirmed chemical bonding between rGO and PANI. It was found that the amide bonds primarily were responsible for conjugation of rGO and PANI. 

The results demonstrated that the rGO/PANI composite is an efficient adsorbent for the AB129. The maximum capacity of this material was determined (using Langmuir isotherm) to be 25.57 mg/g, whereas the adsorption–desorption equilibrium was reached after 40 mins. The adsorption process followed the pseudo-second-order kinetic model. This study proves that the rGO/PANI composite can be successfully used for the adsorption of Acid Blue 129 and other contaminants with similar chemical structure.

## Figures and Tables

**Table 1:** Conditions for rGO/PANI composite preparation.

Time (h)	Temperature (°C)		
	0	50	100
1	rGO/PANI-0,1	rGO/PANI-50,1	rGO/PANI-100,1
8	rGO/PANI-0,8	rGO/PANI-50,8	rGO/PANI-100,8
24	rGO/PANI-0,24	rGO/PANI-50,24	rGO/PANI-100,24

**Table 2:** Comparison of different adsorbents for Acid Blue 129 removal.

Adsorbent	*q*_max_ (mg/g)	Adsorbent Concentration (g/L)	Equilibrium Time (min)	Specific Surface Area (BET) (m^2^/g)	Reference
Activated carbon cloth	61.64	1.4	500	1870	[63]
Almond shell	11.95	16	14	-	[64]
CuO-NP-AC	65.36	0.9	20–25	-	[65]
HCl-Modified Bentonite	13.8	3.33	55	87	[66]
Magnesium-Modified Bentonite	10.8	3.33	40	1310 *	[67]
Iron oxide/carbon nanocomposites	83.42	1	120	695	[68]
rGO/PANI-1,0	25.57	0.5	40	36	This work

* Langmuir surface area.

## Supplementary Materials

The following are available online at https://www.mdpi.com/2073-4360/12/5/1051/s1, Figure S1: SEM image of PANI. Figure S2: SEM images of (a) rGO/PANI-50,1 (b) rGO/PANI-50,24, (c) rGO/PANI-100,24 composites. Table S1: R^2^ values for kinetics experiments of rGO/PANI composite for pseudo-first-order (1st) and pseudo-second-order (2nd) kinetic model.
