# Supplementary material for: A Polymeric Composite Material (rGO/PANI) for Acid Blue 129 Adsorption
Source: Polymers (Basel). 2020 May 3;12(5):1051. doi: 10.3390/polym12051051 (PMC7285098; doi:10.3390/polym12051051)
Supplement: Supplementary file 1 [file polymers-12-01051-s001.pdf]

polymers

Type of the Paper (Article, Review, Communication, etc.)

A Polymeric Composite Material (rGO/PANI) for Acid Blue 129 Adsorption

Tomasz Kukulski [1], Stanisław Wacławek [2,*], Daniele Silvestri [2,*], Kamil Krawczyk [2], Vinod V. T. Padil [2], Ryszard Fryczkowski [1], Jarosław Janicki [1] and Miroslav Černík [2]

[1] Institute of Textile Engineering and Polymer Materials, University of Bielsko-Biala, Willowa 2, 43-309 Bielsko-Biala, Poland; tkukulski@ath.bielsko.pl (T.K.); rfryczkowski@ath.bielsko.pl (R.F.); jjanicki@ath.bielsko.pl (J.J.)

[2] Institute for Nanomaterials, Advanced Technologies and Innovation, Technical University of Liberec, Studentská 1402/2, 46117 Liberec 1, Czech Republic; kamil.krawczyk@tul.cz (K.K.); vinod.padil@tul.cz (V.V.T.P.); miroslav.cernik@tul.cz (M. Č.)

* Correspondence: stanislaw.waclawek@tul.cz (S.W.); daniele.silvestri@tul.cz (D.S.)

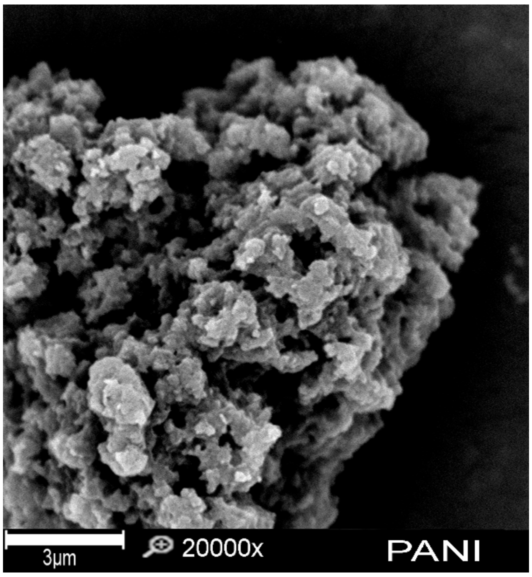

Figure S1. SEM image of PANI (scale bar represents 3 μm).

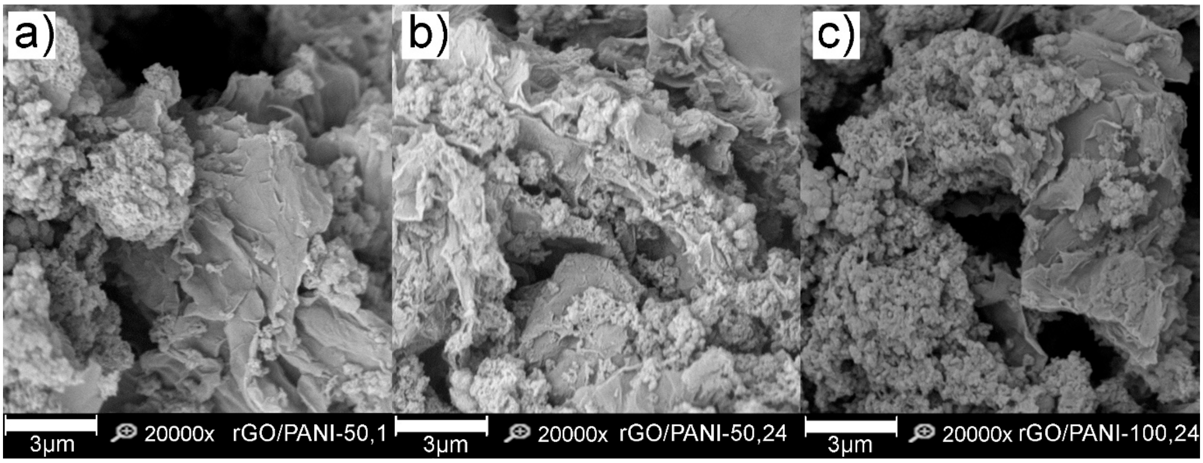

Figure S2. SEM images of a) rGO/PANI-50,1 b) rGO/PANI-50,24, c) rGO/PANI-100,24 composites (scale bar represents 3 μm).

Table S1. R^2 values for kinetics experiments of rGO/PANI composite for pseudo-first-order (1st) and pseudo-second-order (2nd) kinetic model.

Time (h)	Temperature (°C)		
	0	50	100
1	1st 0.939	1st 0.781	1st 0.709
	2nd 0.999	2nd 0.990	2nd 0.990
8	1st 0.511	1st 0.277	1st 0.574
	2nd 0.998	2nd 0.908	2nd 0.998
24	1st 0.398	1st 0.829	1st 0.595
	2nd 0.998	2nd 0.999	2nd 0.999

© 2020 by the authors. Submitted for possible open access publication under the terms and conditions of the Creative Commons Attribution (CC BY) license (http://creativecommons.org/licenses/by/4.0/).
